# Synergism and Antagonism between *Bacillus thuringiensis* Vip3A and Cry1 Proteins in *Heliothis virescens*, *Diatraea saccharalis* and *Spodoptera frugiperda*

**DOI:** 10.1371/journal.pone.0107196

**Published:** 2014-10-02

**Authors:** Ana Rita Nunes Lemes, Camila Chiaradia Davolos, Paula Cristina Brunini Crialesi Legori, Odair Aparecido Fernandes, Juan Ferré, Manoel Victor Franco Lemos, Janete Apparecida Desiderio

**Affiliations:** 1 Faculdade de Ciências Agrárias e Veterinárias, UNESP Univ Estadual Paulista, Departamento de Biologia Aplicada à Agropecuária, Jaboticabal, São Paulo, Brazil; 2 Departamento de Fitossanidade, Jaboticabal, São Paulo, Brazil; 3 Department of Genetics, University of Valencia, Burjassot (Valencia), Spain; Loyola University Medical Center, United States of America

## Abstract

Second generation *Bt* crops (insect resistant crops carrying *Bacillus thuringiensis* genes) combine more than one gene that codes for insecticidal proteins in the same plant to provide better control of agricultural pests. Some of the new combinations involve co-expression of *cry* and *vip* genes. Because Cry and Vip proteins have different midgut targets and possibly different mechanisms of toxicity, it is important to evaluate possible synergistic or antagonistic interactions between these two classes of toxins. Three members of the Cry1 class of proteins and three from the Vip3A class were tested against *Heliothis virescens* for possible interactions. At the level of LC_50_, Cry1Ac was the most active protein, whereas the rest of proteins tested were similarly active. However, at the level of LC_90_, Cry1Aa and Cry1Ca were the least active proteins, and Cry1Ac and Vip3A proteins were not significantly different. Under the experimental conditions used in this study, we found an antagonistic effect of Cry1Ca with the three Vip3A proteins. The interaction between Cry1Ca and Vip3Aa was also tested on two other species of Lepidoptera. Whereas antagonism was observed in *Spodoptera frugiperda*, synergism was found in *Diatraea saccharalis*. In all cases, the interaction between Vip3A and Cry1 proteins was more evident at the LC_90_ level than at the LC_50_ level. The fact that the same combination of proteins may result in a synergistic or an antagonistic interaction may be an indication that there are different types of interactions within the host, depending on the insect species tested.

## Introduction

Bioinsecticides based on *Bacillus thuringiensis* Berliner are an alternative to chemical insecticides to control important agricultural pests and account for almost 95% of the total sales within the biopesticide market [1]. One important application of the genes that encode insecticidal proteins from this bacterium is their introduction into plants, which has allowed for the development of transgenic plants that are resistant or tolerant to insect pests.

*Bacillus thuringiensis* produces two major categories of active proteins, Cry and Vip. Cry proteins (encoded by *cry* genes) are produced during the sporulation phase of the bacterium. More than 600 *cry* genes have been described (http://www.lifesci.sussex.ac.uk/home/Neil_Crickmore/Bt/toxins2.html). The Cry proteins are used worldwide for insect control, and their mode of action has been the subject of many studies and can be considered rather well characterized. Vip proteins are produced during the vegetative growth phase of the bacterium and are secreted into the culture medium and have no sequence homology with Cry proteins [2]. They are classified into four groups (http://www.lifesci.sussex.ac.uk/home/Neil_Crickmore/Bt/vip.html). Vip1 and Vip2 act as a binary protein with activity against coleopteran larvae [3]–[4], Vip3 proteins are active against a large number of economically important lepidopteran pests [5]–[6] and, so far, very little is known about Vip4 proteins. Some studies have shown that the transcription of several *cry* genes in *B. thuringiensis* occurs before the onset of sporulation [7] and, therefore, at least some Cry and Vip toxins may be expressed together and interact within the insect gut.

The use of *cry* genes in transgenic plants became a strategy of pest control in the mid-1990s. The first generation of insect resistant plants (*Bt*-crops) was generated by transferring a single *cry* gene into the genome of the plant [8]–[9]–[10]. Although very effective, a challenge associated with this type of plant is the potential for the target pests to develop resistance against the single insecticidal protein that is produced by the plant. Resistance to Cry proteins has been found in different pests that have evolved resistance under laboratory selection [11] and, more recently, in the field against plants that express single *cry* genes [12]. The development of resistance to Cry toxins is a process that is governed by a large number of interacting factors, including reduced proteolytic activity in the insect's gut, and changes in the membrane receptor [11]. Second- generation *Bt*-crops incorporate a combination of two or more *B. thuringiensis* genes into plants, offering producers a broader spectrum of action and reducing the chances of target pests developing resistance [13]. An example of a second generation *Bt*-crop is the second most widely adopted insect-resistant crop, *Bt* cotton [14]. This crop was first developed to express a single gene (*cry1Ac*). Currently, approved second-generation cotton contains several combinations of *B. thuringiensis* genes, such as Bollgard II (expressing Cry1Ac and Cry2Ab) [15], WideStrike (expressing Cry1Ac and Cry1F) (http://www.dowagro.com/phytogen/widestrike/) and VipCot (expressing Cry1Ab and Vip3A) [16].

The choice of which genes to use in these combinations is mainly driven by their complementarity and is based on the ability of the two toxins to bind to different receptors within the insect gut because binding-site alteration has been shown to be a widespread mechanism of resistance [17]–[18]–[19]. However, another important aspect that has not received much attention is the possibility of interaction effects between proteins, which can be either synergistic or antagonistic [20]. Synergism enhances the control efficiency, allowing the use of lower doses of each component of the mixture [21]. Conversely, if the components of the mixture have antagonistic effects, then the potential advantages of using genes in combination would be counteracted by the decrease in effectiveness. These types of interactions can be assessed by evaluating the toxicity of toxin mixtures vs. that of individual toxins. The selection of a suitable pair of insecticidal proteins to be co-expressed in the same plant should take into account, not only differences in the mode of action, but also potential interactions between these proteins.

In the present study, we aimed to determine possible interactions between three members of the Cry1 class of proteins (Cry1Aa, Cry1Ac and Cry1Ca) and three members of the Vip3A class of proteins (Vip3Aa, Vip3Ae and Vip3Af) in *Heliothis virescens* (Fabr.) (Lepidoptera: Noctuidae). This pest caused extensive damage to cotton fields in the USA prior to the commercialization of Bt cotton. During the larval stage, this pest is able to completely destroy the tissues of different plant organs. It has been estimated that one larva can be responsible for causing damage to more than 15 productive structures within a plant during its larval cycle, decreasing the production of cotton. Indirect damage is very common in damaged plants due to the penetration of microorganisms through the holes the larvae make [22].

Because of the antagonism found between Cry1Ca and Vip3Aa in *H. virescens*, we extended this study to include two important Brazilian pests, *Diatraea saccharalis* (Fabr.) (Lepidoptera: Pyralidae) and *Spodoptera frugiperda* (Smith) (Lepidoptera: Noctuidae). The former, also known as the sugarcane borer, is the insect pest that causes the most damage to sugarcane crops. Its feeding behavior, which is characterized by the larva's penetration into the plant stems, limits the control of this insect by conventional methods [23]. The armyworm, *Spodoptera frugiperda*, is a polyphagous species that is autochthonous to the tropical regions of the South American continent. In Brazil this species is one of the most devastating to maize and is an economically important insect pest, causing damage to other crops, including soybean, cotton, rice, sorghum and vegetables [24].

The results of the overall study indicate that the same combination of proteins may result in a synergistic or an antagonistic interaction, depending on the insect species tested.

## Materials and Methods

### Insect colonies

Eggs from the three lepidopteran species tested were kindly supplied by different Brazilian institutions where the populations have been established and maintained for more than 10 years. *H. virescens* was obtained from the LBI (ESALQ/USP) Piracicaba, SP, *S. frugiperda* was obtained from APECOLAB (UNESP-FCAV) Jaboticabal, SP, and *D. saccharalis* was obtained from São Martinho Sugar Mill Biocontrol Lab., Pradópolis, SP, Brazil.

### Protein source and preparation

The recombinant *Escherichia coli* XL-1 Blue clones expressing just one type of Cry protein used in this study were kindly supplied by Ruud de Maagd (Plant Research International, Wageningen, Netherlands). Cry1Aa, Cry1Ac and Cry1Ca proteins were expressed as protoxin inclusion bodies. Cry protoxins were used directly after solubilization from the inclusion bodies in buffer (50 mM NaHCO_3_ pH 10.0; 100 mM NaCl; 10 mM DTT), as previously described [25].

*E. coli* clones carrying the genes encoding Vip3Aa, Vip3Ae and Vip3Af were kindly provided by Bayer CropScience (Ghent, Belgium) and were expressed in *E. coli* WK6. Protein expression was accomplished by following the protocol of Chakroun et al. [26], with a change in the amount of isopropyl-D-thiogalactopyranoside (IPTG) to a final concentration of 1 mM. Vip3A protoxins were used directly from the supernatant after centrifugation and filtration of the cell lysate (in phosphate buffer 20 mM pH 7.4; 0.5 M NaCl).

**Figure 1:**
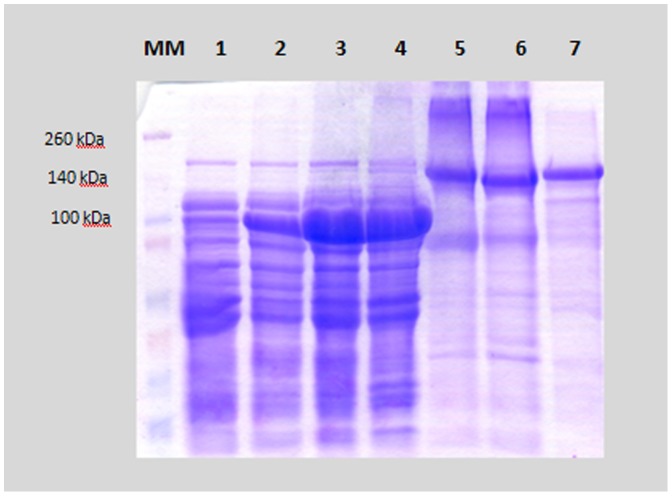
SDS-PAGE of *Escherichia coli* lysates. MM, Molecular Mass Marker “Spectra TM Multicolor Broad Range Protein Ladder” (Fermentas); lane 1, Vip3A not induced with IPTG; lane 2, Vip3Aa; lane 3, Vip3Ae; lane 4, Vip3Af lane 5, Cry1Aa; lane 6, Cry1Ac; lane 7, Cry1Ca.

Protein expression from each clone was verified by sodium dodecyl sulfate polyacrylamide gel electrophoresis (SDS–PAGE). In the induced clones, Cry1 and Vip3A proteins showed bands of molecular weights of approximately 135 kDa and 85 kDa, respectively (Fig. 1). The concentration of Cry1 and Vip3A proteins in the preparations were determined by densitometry of the SDS-PAGE gels using bovine serum albumin (BSA) as the standard with Bionumerics software (Applied-Maths).

### Bioassays

Toxicity was evaluated by applying the protoxin preparations on the surface of the artificial diet, which was dispensed on 2 cm^2^ wells in polystyrene trays (“Cell Wells, Corning Glass Works”, Corning, New York). Artificial diets for *H. virescens*, *S. frugiperda* and *D. saccharalis* were prepared according to published methods [27]–[28]–[29]. Sixteen neonate larvae were used per concentration and each experiment was repeated three times (a total of 48 larvae per concentration). Trays were kept at 25±2°C with a relative humidity of 70±10% and a 14∶10 h (light∶dark) photoperiod. Mortality was recorded at five days. Either distilled water or the solubilization buffers were used instead of the proteins as controls for natural mortality. POLO-PC software (LeOra software, Berkeley, CA) was used to estimate LC_50_ and LC_90_ values in dose-response bioassays and to obtain chi-square values.

### Tests for Cry1 and Vip3A interactions

All tests to compare observed and expected values were conducted simultaneously. An initial test to search for interactions between Cry1 and Vip3A proteins was performed at a single concentration of each protein. The concentration of each toxin in the mixture was selected so that it was that of their respective LC_50_ value. The expected mortality in the absence of interactions was estimated assuming the hypothesis of simple independent action [30]. Under this hypothesis, the proportion (*P*) of larvae dying from exposure to a mixture of two toxins was calculated as:
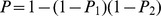
where *P*_1_ and *P*_2_ represent the proportions of dead larvae for toxins 1 and 2, respectively. This formula is equivalent to equation 11.33 of Finney [30]. We used the observed mortality values obtained at the theoretical LC_50_ value with single toxins to calculate the expected mortality of toxin mixtures. Significance of deviations between expected and observed mortality was determined by using Fisher's exact test.

A second test for interactions was performed using concentration-response assays in which the proportions of the two proteins in the mixture matched the ratio between their respective LC_50_ values. The expected mortality in the absence of interactions was estimated assuming the hypothesis of simple similar action [30] using the formula of Tabashnik (20), which derives from equation 11.8 of Finney [30]:
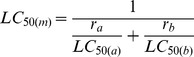
where LC_50(*m*)_ is the median lethal concentration of the mixture, LC_50(*a*)_ and LC_50(*b*)_ are the respective median lethal concentrations of the individual components, and *r_a_* and *r_b_* are the relative proportions of the *a* and *b* components in the mixture. The same formula was used to determine the interaction between the proteins at the LC_90_ level. The antagonism factor (AF) was determined by the ratio of the observed LC_50_ over the expected LC_50_. An AF value larger than 1 indicates an antagonistic interaction, a value of 1 indicates additive toxicity, and a value lower than 1 indicates a synergistic interaction. The antagonism factor is related to the synergism factor (SF) by AF = 1/SF. The interaction was considered significant if the expected value did not fall within the fiducial limits of the respective observed value.

## Results

### Insecticidal activity against *H. virescens*

Concentration–response assays were performed to determine the LC_50_ and LC_90_ values of the six protoxins, and the results are shown in Table 1. Among Cry1 proteins, Cry1Ac was the most toxic, with the LC_50_ values approximately 80-fold lower than those of Cry1Aa and Cry1Ca; at the level of LC_90_, these differences were maintained or slightly increased. Regarding Vip3A proteins, similar LC_50_ and LC_90_ values were obtained for each of the three proteins. Compared to Cry1 proteins, Vip3Ae and Vip3Af were slightly more active than Cry1Aa and Cry1Ca proteins (approximately 4-fold), but less active than Cry1Ac (20 to 40-fold) at the level of LC_50_. However, at the level of LC_90_, the three Vip3A proteins were considerably more active than Cry1Aa and Cry1Ca (from 8 to 30-fold) and not significantly different from Cry1Ac. This difference in relative activity at the LC_50_ and LC_90_ levels is due to the difference in the respective slopes of regression lines between Cry1 and Vip3A proteins. Negative controls did not cause mortality under the conditions of the assays.

**Table 1:** Susceptibility of *H. virescens* neonate larvae to Cry1 and Vip3A protoxins.^a^

Protein	b ± SE^b^	Chi-Square	LC_50_ (FL min – max)^c^	LC_90_ (FL min – max)^c^
Cry1Aa	1.03±0.16	15.007	3.50 (1.63–11.9)	61.6 (16.2–1620)
Cry1Ac	1.38±0.22	11.916	0.040 (0.021–0.110)	0.34 (0.12–5.44)
Cry1Ca	1.35±0.22	10.771	3.10 (1.74–5.51)	27.5 (11.2–233)
Vip3Aa	4.07±0.67	6.6642	1.65 (1.28–2.34)	3.41 (2.39–6.35)
Vip3Ae	2.36±0.33	12.508	0.946 (0.725–1.289)	3.30 (2.16–7. 08)
Vip3Af	3.57±0.68	4.6937	0.874 (0.708–1.026)	2.00 (1.59–3.05)

a Values represent the mean from three replicates of 16 larvae per replicate (n = 48).

b Slope ± standard error.

c Values are expressed as µg/cm^2^ with 95% fiducial limits (at 5 days).

### Search for interactions between Vip3A and Cry1 proteins in *H. virescens*

Various combinations between Vip3A proteins and Cry1 proteins were tested at a single concentration (that of their respective LC_50_ values), and observed mortality was compared to the expected mortality assuming no interaction (Table 2). Significant interactions were found with the combination of Cry1Ca with each of the three Vip3A proteins and of Vip3Af with all three of the Cry1 proteins (P<0.05). In all cases, the interaction was negative (i.e., antagonistic). The highest antagonistic effect was observed with Cry1Ca and Vip3A combinations.

**Table 2:** Susceptibility of *H. virescens* neonate larvae to combinations of Vip3A and Cry1 protoxins.

Protein combination	Respective Concentrations^a^	Mortality (%)		Fisher's exact test^d^	Chi-square (P)^e^
		Observed^b^	Expected^c^		
Cry1Aa	3.50	44	50		
Cry1Ac	0.04	52	50		
Cry1Ca	3.10	42	50		
Vip3Aa	1.65	52	50		
Vip3Ae	0.95	50	50		
Vip3Af	0.87	50	50		
Vip3Aa+Cry1Aa	1.65+3.50	69	73	0.4113	0.2017 (0.6534)
Vip3Aa+Cry1Ac	1.65+0.04	67	77	0.1820	1.2882 (0.2564)
Vip3Aa+Cry1Ca	1.65+3.10	33	72	0.00009**	15.1007 (0.0001**)
Vip3Ae+Cry1Aa	0.946+3.50	73	72	0.5907	0.0000 (1.0000)
Vip3Ae+Cry1Ac	0.946+0.04	63	76	0.1354	1.7455 (0.1864)
Vip3Ae+Cry1Ca	0.946+3.10	31	71	0.0001**	15.0482 (0.0001**)
Vip3Af+Cry1Aa	0.874+3.50	50	72	0.0177*	5.3211 (0.0211*)
Vip3Af+Cry1Ac	0.874+0.04	50	76	0.0149*	5.7501 (0.0165*)
Vip3Af+Cry1Ca	0.874+3.10	37	71	0.0009**	10.7413 (0.0010**)

a Concentrations of proteins were chosen such as to equal their respective LC_50_ values. Values are expressed as µg/cm^2^.

b Each value represents the mean from three replicates of 16 larvae per replicate (n = 48).

c Expected mortality considering simple independent action.

d Asterisks indicate significant differences at P<0.05, and two asterisks at P<0.001.

e Chi-square and P values.

### Analysis of antagonism in *H. virescens* with concentration-response assays

The combinations that showed antagonism in the previous analysis were investigated further by concentration-response analyses. The proportions of the two proteins used in the mixtures were such that they matched the ratio between their respective LC_50_ values. The observed LC_50_ and LC_90_ values were compared to the expected values assuming no interaction (Table 3). At the level of LC_50_, moderate antagonism was observed in all combinations with Cry1Ca (AF = 6 to 9), and only a slight or even non-significant antagonism of Vip3Af with Cry1Aa and Cry1Ac. At the level of LC_90_, the antagonism factor increased for all combinations, especially for those involving Cry1Ca (AF = 23 to 59).

**Table 3:** Evaluation of antagonism, at the LC_50_ and LC_90_ level, of combinations of Vip3A and Cry1 protoxins to *H. virescens* neonate larvae.

Protein combinations	Proportions^b^	b ± SE^c^	Chi-square	LC_50_ (µg/cm^2^)^a^			LC_90_ (µg/cm^2^)^a^		
				Observed (FL_95_)^d^	Expected^e^	AF^f^	Observed (FL_95_)^d^	Expected^e^	AF^f^
Vip3Aa+Cry1Ca	1∶2	1.29±0.22	2.6125	19.1 (14.0–29.9)	2.40	8.0	187 (88–782)	8.20	22.8
Vip3Ae+Cry1Ca	1∶3	0.86±0.21	0.3782	18.7 (10.3–77.5)	1.98	9.4	571 (115–50850)	9.71	58.8
Vip3Af+Cry1Aa	1∶3	1.60±0.15	3.3717	2.72 (2.16–3.43)	2.00	1.4	17.2 (11.9–28.6)	7.29	2.4
Vip3Af+Cry1Ac	25∶1	1.11±0.14	7.3582	1.25 (0.43–5.10)	0.49	NS	17.7 (4.6–4140)	1.68	10.5
Vip3Af+Cry1Ca	1∶3	1.07±0.21	1.5602	11.7 (7.7–24.8)	1.89	6.2	186 (63–2060)	6.57	28.3

a Each data point was obtained from three replicates of 16 larvae per replicate (n = 48).

b Proportions of proteins were chosen approximately to match those of Table 2.

c Slope ± standard error.

d FL_95_: 95% fiducial limits.

e Expected mortality considering simple similar action.

f AF: Antagonism factor, calculated as the ratio of the observed LC_50_ over the expected LC_50_. NS = not significant.

### Interaction of Vip3Aa and Cry1Ca in *D. saccharalis* and *S. frugiperda*

Because Cry1Ca was the protein which showed the strongest antagonism with Vip3A proteins, the combination Vip3Aa+Cry1Ca was tested for interactions in two additional lepidopteran species at a 1∶2 ratio, which was the ratio that revealed antagonism in *H. virescens* (Table 4). For *D. saccharalis*, both proteins were equally active at the level of LC_50_, however, due to the difference in regression slopes, the activity of Cry1Ca was 15-fold greater than Vip3Aa at the level of LC_90_. This protein combination yielded much lower LC_50_ and LC_90_ values than expected, with antagonism factors lower than 1, indicating synergism instead of antagonism. The estimated synergism factors (SF = 1/AF) were 14 and 9, respectively.

**Table 4:** Susceptibility of *D. saccharalis* and *Spodoptera frugiperda* neonate larvae to Vip3Aa and Cry1Ca protoxins and their 1: 2 combination.

Insect species	Proportions	b ± SE^a^	LC_50_ (µg/cm^2^)^b^			LC_90_ (µg/cm^2^)^b^		
	Vip3Aa∶Cry1Ca		Observed (FL_95_)^c^	Expected^d^	AF^e^	Observed (FL_95_)^c^	Expected^d^	AF^e^
*D. saccharalis*	1∶0	0.55±0.07	0.13 (0.07–0.24)			27.1 (7.87–129)		
	0∶1	1.60±0.16	0.28 (0.19–0.40)			1.78 (1.13–3.57)		
	1∶2	0.98±0.10	0.014 (0.010–0.019)	0.20	0.070	0.28 (0.16–0.63)	2.58	0.11
*S. frugiperda*	1∶0	1.53±0.22	0.44 (0.32–0.65)			3.03 (1.69–8.12)		
	0∶1	1.43±0.25	0.052 (0.034–0.084)			0.41 (0.21–1.47)		
	1∶2	0.65±0.54	0.30 (0.13–0.72)	0.074	4.05	27.6 (7.21–277)	0.58	47.6

a Slope ± standard error.

b Each data point was obtained from three replicates of 16 larvae per replicate (n = 48).

c FL_95_: 95% fiducial limits.

d Expected mortality considering simple similar action.

e AF: Antagonism factor.

The activity of Cry1Ca was approximately 8-fold higher than that of Vip3Aa for *S. frugiperda*. In contrast to *D. saccharalis*, the combination of these two proteins in *S. frugiperda* was antagonistic, being much more notable at the level of LC_90_.

## Discussion

Bioassays to determine the potency of *B. thuringiensis* insecticidal proteins are usually performed with individual insecticidal proteins, and this is useful to estimate their theoretical contribution to pest control when in toxin mixtures (either in sprays or expressed within *Bt*-crops). Nevertheless, it is known that some combinations of insecticidal proteins may have synergistic or antagonistic effects [31]–[32]–[33]. Therefore, when choosing combinations of genes coding for insecticidal proteins to be expressed in plants, it is not only wise to consider differences in their mode of action but also to consider possible interactions among insecticidal proteins.

Among the proteins tested in the present study, Cry1Ac was the most active protein against *H. virescens*. Vip3A proteins were over 20-fold less active than Cry1Ac at the level of LC_50_; however, at the level of LC_90_, the differences between Cry1Ac and the three Vip3A proteins were not significant (considering the overlap in the fiducial limits). The differences observed when comparing the results at the LC_50_ level vs. the LC_90_ level are a consequence of the differences in the slopes of the dose-mortality regression lines between Cry1 (1.03 to 1.38) and Vip3A (2.36 to 4.07) proteins (Table 1). According to the LC_50_ values, Vip3A proteins require higher doses to be effective against the insects, but once a critical threshold is reached, the response increases rapidly with the dose. In contrast, Cry1 proteins show a more common dose-mortality response, represented by a shallower slope. One interpretation of the high slope values of Vip3A proteins could be that this type of protein requires a threshold concentration in the insect midgut.

Our data are generally in agreement with those reported by other authors. In a study that evaluated the toxicity of Cry1Aa and Cry1Ac to *H. virescens* from Louisiana (USA), the former was found to be 10-fold less toxic than the latter (LC_50_ values of 5 ng/cm^2^ and 0.5 ng/cm^2^, respectively) [34]. Van Rie et al. [35] estimated an LC_50_ value of 90 ng/cm^2^ for Cry1Aa, which is 18 times lower than that found by Jurat-Fuentes and Adang [34] and closer to the value found in the present study. In the same study, the LC_50_ value for Cry1C could not be estimated because of its low toxicity (the LC_50_ value was greater than the highest concentration tested: 1875 ng/cm^2^) [35], which is in agreement with our results.

The toxicity of the Vip3A proteins used in this study against *H. virescens* showed no significant differences. Although these proteins were less active than Cry1Ac at the LC_50_ level, it is important to note that at the LC_90_ level they all were considerably more active than Cry1Aa and Cry1Ca and not significantly different from Cry1Ac.

The LC_50_ values obtained for the Vip3A proteins with *S. frugiperda* in this study are substantially higher than values published elsewhere [26]–[31]–[36]–[37], most likely because the mortality in the present study was scored earlier (5 d vs. 7 d in the others) and the mortality of *S. frugiperda* is known to be dependent on the length of the bioassay [26]. Other differences in bioassay results may arise from differences in the source and purity of the insecticidal proteins, as well as in the insect colonies employed. One way to overcome problems in methodology when comparing insecticidal activities is to compare activity ratios within laboratories. Based on the very few studies performed that compare the activity of Cry1Ac and Vip3A proteins, our results are in agreement with those reported by Jackson et al. with *H. virescens* [38]; in both cases, LC_50_ values differ by a factor of approximately 100-fold.

The efficiency of a mixture should not be solely estimated based on the efficacy of the individual ingredients, but on the synergism between their components [39]. When a mixture is more toxic than expected, the interaction between the components is considered to be synergistic, when the mixture is less toxic than expected, it indicates an antagonistic interaction [30]. Interactions between Vip3Aa and Cry1 proteins have recently been found in several insect species. A recent study in our laboratory, which investigated the interaction between Vip3Aa and Cry1Ia, revealed synergism of these two proteins in *S. frugiperda* (SF = 6.4), *Spodoptera albula* (Lepidoptera: Noctuidae) (SF = 4.2) and *Spodoptera cosmioides* (Lepidoptera: Noctuidae) (SF = 4.1), and a very slight antagonism in *Spodoptera eridania* (Lepidoptera: Noctuidae) (SF = 0.3) [31]. In another study, the combination of Vip3Aa and Cry9Ca was found to be slightly synergistic (SF = 1.4) in *Plutella xylostella* (Lepidoptera: Plutellidae) [32]. Interactions between Vip3Aa and another protein class (Cyt2Aa) were studied in five insect pests. Slight synergistic effects were found in *Chilo suppressalis* (Lepidoptera: Pyralidae) and *Spodoptera exigua* (Lepidoptera: Noctuidae). No synergism was found in *Chironomus tepperi* (Diptera: Chironomidae) and *Helicoverpa armigera* (Lepidoptera: Noctuidae), and a slight antagonistic effect was found in *Culex quinquefasciatus* (Diptera: Culicidae) [33].

In the present study, we have found antagonism between Vip3A and Cry1 toxins in *H. virescens*. All Vip3A proteins tested, when combined with Cry1Ca, presented a clear antagonistic effect, and just a very slight antagonism was observed for the combinations of Vip3Af with Cry1Aa (almost negligible) and Vip3Af with Cry1Ac (only significant at the LC_90_ level) (Table 3). The antagonism factor was higher, in all cases, at the LC_90_ level than at the LC_50_ level. This difference seems to be related to the LC differences between Cry1Ca and Vip3A, which are much higher at the LC_90_ level (8 to 14-fold) than at the LC_50_ level (2 to 3-fold). To some extent, the higher the difference is between LC values, the more conspicuous the antagonistic effect.

Antagonism between Vip3Aa and Cry1Ca was also detected in *S. frugiperda* (AF rations ranged from 4 to 48 at the LC_50_ and LC_90_ level, respectively). However, this same combination resulted in synergism in *D. saccharalis*. It is worth noting that, in all cases, the interaction (either antagonistic or synergistic) between Vip3A and Cry1 proteins was more remarkable at the LC_90_ than at the LC_50_ level. If these interactions are maintained under field conditions (either in plants or in sprays), our results indicate that, at the doses used to control a pest (which are near the LC_90_ value or higher), the effect of the interaction between toxins should be even stronger. The fact that the same combination of proteins may result in a synergistic or an antagonistic interaction might indicate different modes of action depending on the insect species considered.

The mechanism related to the observed antagonism is not known. It is possible that the proteins may physically interact in a way in which they sequester each other, forming a complex that yields both proteins inactive. Alternatively, the formation of the complex could just mask an epitope in the most toxic protein, preventing it from interacting with the membrane receptor. The antagonism could also result from steric interactions, where both toxins bind to different epitopes in the same membrane molecule [23]–[40]. It would be worthwhile to test these and similar hypotheses to shed light on the antagonism between Cry1C and Vip3 that was observed in *H. virescens* and *S. frugiperda*. Regarding the mechanisms to explain synergism, several hypotheses have been proposed. One hypothesis is that Cry1 proteins may form hetero-oligomers that have a better ability to insert into the membrane than the corresponding homo-oligomers, resulting in greater toxicity against their target pests [41]. However, in the case of Vip3 proteins, oligomerization has never been reported, making it very unlikely that they could form hetero-oligomers with Cry1 proteins. A more plausible explanation is that the two proteins induce larger pores in the larval midgut membrane than when either protein does individually. In this respect, Lee et al. [40] demonstrated that Cry1Ac and Cry1Aa toxins showed synergistic activity against *Lymantria dispar* (Lepidoptera: Lymantriidae) caterpillars, and also found that the combination of the two toxins led to the formation of larger pores than when using the toxins individually. Interestingly, the authors reported antagonism of the Cry1Aa+Cry1Ab combination.

We are aware that our results have been obtained under laboratory conditions (in the absence of predators and parasitoids), using artificial diet (in the absence of phytochemicals) and measuring mortality at 5 days (not considering growth arrest). To confirm whether the interactions found in this study would persist or even increase under field conditions, the protein pairs showing interactions under laboratory conditions should be further tested where both are expressed in the same plant. Nevertheless, the results presented here, along with those obtained by other authors on the interactions among insecticidal proteins in different insect pests, clearly point to the importance of this type of studies and, thus, they should be considered complementary to studies dealing with the mode of action of different toxins when selecting the appropriate choice of gene combinations for pyramided *Bt*-crops.
